# Changes in Psychiatric Inpatient Service Utilization During the First and Second Waves of the COVID-19 Pandemic

**DOI:** 10.3389/fpsyt.2022.829374

**Published:** 2022-02-17

**Authors:** Matilda Hamlin, Thérèse Ymerson, Hanne Krage Carlsen, Marzia Dellepiane, Örjan Falk, Michael Ioannou, Steinn Steingrimsson

**Affiliations:** ^1^Institute of Neuroscience and Physiology, Sahlgrenska Academy, University of Gothenburg, Gothenburg, Sweden; ^2^Region Västra Götaland, Department of Psychiatry, Sahlgrenska University Hospital, Gothenburg, Sweden; ^3^Department of Occupational and Environmental Medicine, School of Public Health and Community Medicine, Institute of Medicine, Sahlgrenska Academy, University of Gothenburg, Gothenburg, Sweden

**Keywords:** COVID-19, acute psychiatric services, psychiatric admission, digital psychiatry, mental health

## Abstract

The COVID-19 pandemic has caused societal restrictions and public fear which may have impacted the pattern of seeking psychiatric care. There has generally been a decrease in the numbers seeking acute psychiatric care. It is important to investigate which groups seeking psychiatric treatment have decreased in number. The aim of our investigation was to identify which groups have a changed pattern in acute psychiatric service utilization during the first two waves of the COVID-19 pandemic. The study investigated changes in the rate and pattern of visits and hospital admissions for psychiatric disorders at a large Swedish hospital. A register-based study was conducted using administrative data on adult psychiatric emergency department visits (PEVs) and hospital admission rates. Data during the first two COVID-19 waves were compared to corresponding control periods in 2018–2019. Furthermore, a survey was performed among patients visiting the Psychiatric Emergency Department on their views of COVID-19 and acute psychiatric care. During the COVID-19 periods, PEVs were reduced overall by 16 and 15% during the first and second wave, respectively (*p* < 0.001 in both cases), while the rate of admissions remained unaltered. PEVs were significantly reduced for most psychiatric diagnosis subgroups except for patients with schizophrenia and other related psychotic disorders as well as for those who required ongoing outpatient care. Most of the survey respondents disagreed that the pandemic affected their visit and about a quarter thought a video call with a doctor could have replaced their visit. In conclusion, there was a significant reduction in overall PEVs during both COVID-19 waves but this did not affect the numbers requiring admission for psychiatric inpatient care.

## Introduction

In March 2020, the World Health Organization declared the COVID-19 disease as a global pandemic (1). Since then, various societal efforts such as social distancing and restrictions on mobility have been implemented worldwide to mitigate the spread of the COVID-19 virus. These measures along with other directly COVID-19-related factors might be expected to influence those suffering from mental health issues not only during the ongoing pandemic but also in a longer perspective (2). However, it remains to be seen how much COVID-19 has impacted public mental health (3, 4) and, in particular, among persons with previous mental disorders (5). Furthermore, changes in psychiatric health care services during the pandemic may have affected not only care seekers but also the working environment and health care workers (6).

National strategies have differed from restriction of public movements to severe quarantine and lockdown measures. In Sweden, the strategy has consisted of general recommendations of limited mobility (i.e., encouraging people not to travel) and social distancing in contrast to the full lockdown in many other European countries (7).

Acute psychiatric services have been affected during the first wave of the pandemic with a reported 13–56% decrease across countries in adult psychiatric emergency room visits (PEVs) (8–15). A similar decrease in psychiatric admission rates to inpatient services has been reported between 10 and 41% (13, 16, 17). The decreased rates have not been uniform for all groups and, for example in Sweden, higher rates of admission have been reported during the first wave of the COVID-19 pandemic among patients with psychotic disorders and emotionally unstable personality syndrome (12). The underlying mechanisms for reduced rates are largely unknown but, besides limitations in mobility and physical distancing from others, fear of infection may have played a role in the impact on PEVs (18). Furthermore, studies imply that psychiatric patients may have a higher risk of death related to COVID-19 infection (19). There is a lack of studies investigating which groups were at higher risk of being affected during the pandemic and a lack of studies on patient perspectives on psychiatric care during this time.

The aim of this study was to investigate the changes in utilization of acute psychiatric services during the COVID-19 pandemic, with a focus on risk groups defined by age, sex, psychiatric diagnosis, and requirement for ongoing psychiatric outpatient care. PEVs and psychiatric hospital admissions were chosen to identify possible changes among severely ill patients who were in need of in-patient care. In addition, we investigated the patients' own views on how their psychiatric care was affected by the COVID-19 pandemic.

## Materials and Methods

### Study Design

Two different data collections were performed. First, a register-based data collection was conducted using an administrative database for PEVs and admission rates at the psychiatric units at Sahlgrenska University Hospital, Gothenburg, Sweden. Data was collected from 1 January 2018 to 1 April 2021 regarding PEVs and psychiatric admissions. The collected data also included age, sex, psychiatric diagnosis, and requirement for ongoing psychiatric outpatient care. Secondly, an anonymized survey was distributed to patients seeking treatment at the Psychiatric Emergency Department during November and December 2020. The survey was developed for this study and the questions chosen by three specialists in psychiatry (MD, MI, and SS) based on clinical observations during the first months of the COVID-19 pandemic. A revised version was developed in a consensus meeting after feedback from psychiatric personal before distribution. The survey was conducted using a paper form and was available to all patients without any specific exclusion criteria. The first part of the survey gathered demographic information (age, sex, marital status, etc.) and clinical information (reason for contact and whether the contact was voluntarily). The second part included 17 statements concerning patient perceptions of acute psychiatric care in relation to COVID-19. The study was approved by the Swedish Ethical Review Authority (No. 2020-04222).

### Study Setting

The Sahlgrenska University Hospital serves an area of approximately one million adult inhabitants, which makes it one of the largest in Northern Europe (20). The Psychiatric Emergency Department is open 24 h daily all week and is available for all with or without referral. The Swedish health care system is mainly government funded and is universal for all citizens with just a small fee per hospital visit. The organization is built upon a triage system that prioritizes patients into different levels of urgency for a doctor's assessment depending on the severity of symptoms. A nurse specialized in psychiatry can send patients with non-acute symptoms home or refer to outpatient care such as primary care without a doctor's assessment (21). However, during the pandemic, the triage system included a doctor's assessment.

### Measurements

Changes in psychiatric health service utilization were measured as changes in the number of PEVs and hospital admissions. The rate of PEVs and hospital admissions were first analyzed as a total number and then by age, sex, main psychiatric diagnosis, and whether patients had ongoing psychiatric outpatient care. Age and sex were defined by the patient's personal identity number.

#### COVID-19 Wave Periods

Two specified time periods of higher infection rates during the COVID-19 pandemic were defined as first (March 10 to June 14, 2020) and second (October 26, 2020 to March 28, 2021) waves. Data was compared to corresponding time periods in previous years (2018–2019). The pandemic time periods were based on recommendations to “avoid all unnecessary visits to health care” from the Public Health Agency of Sweden (22).

#### PEVs

PEVs were defined as the total number of visits to the Psychiatric Emergency Department when conducting descriptive analysis and were defined as the mean number of visits weekly when conducting linear regression analysis.

#### Psychiatric Admissions

Psychiatric hospital admissions were defined by the registered discharge date but is referred to as hospital admissions in the subsequent text since it is a more frequently used term.

#### Classification of Psychiatric Disorders

The main psychiatric disorders were classified using the International Classification of Diseases version 10 (ICD-10) as follows:

- Substance use disorders = F10–F19
- Schizophrenia and other related psychotic disorders = F20–F29
- Bipolar and related mood disorders = F30–F31
- Unipolar and related mood disorders = F32–F39
- Neurotic, stress-related, and somatoform disorders = F40–F48
- Disorders of personality and behavior in adult persons = F60–F69

#### Survey

In the second data collection, an anonymized survey, a total of 17 statements were each scored on 5-point Likert scales (1 = strongly disagree to 5 = strongly agree), which were thereafter categorized into “disagree” (score 1–2), “neutral” (score 3), and “agree” (score 4–5).

### Statistics

For PEV and admission data, frequencies with 95% confidence interval (CI) and percentages were used to describe the data. Linear regression was used to obtain *p*-values for the change in mean weekly visits or admissions compared to the control period for each COVID-19 pandemic wave. Only variables with at least two visits/admission per week were included. For the survey data, responses were presented as descriptive statistics [percentage and mean with 95% CI] for the 17 different statements included in the survey. Data analyses were performed using R 4.0.2 (23) and IBM SPSS statistics 25. A *p-*value below 0.05 was considered statistically significant.

## Results

### Psychiatric Emergency Department Visits

**Figure 1:**
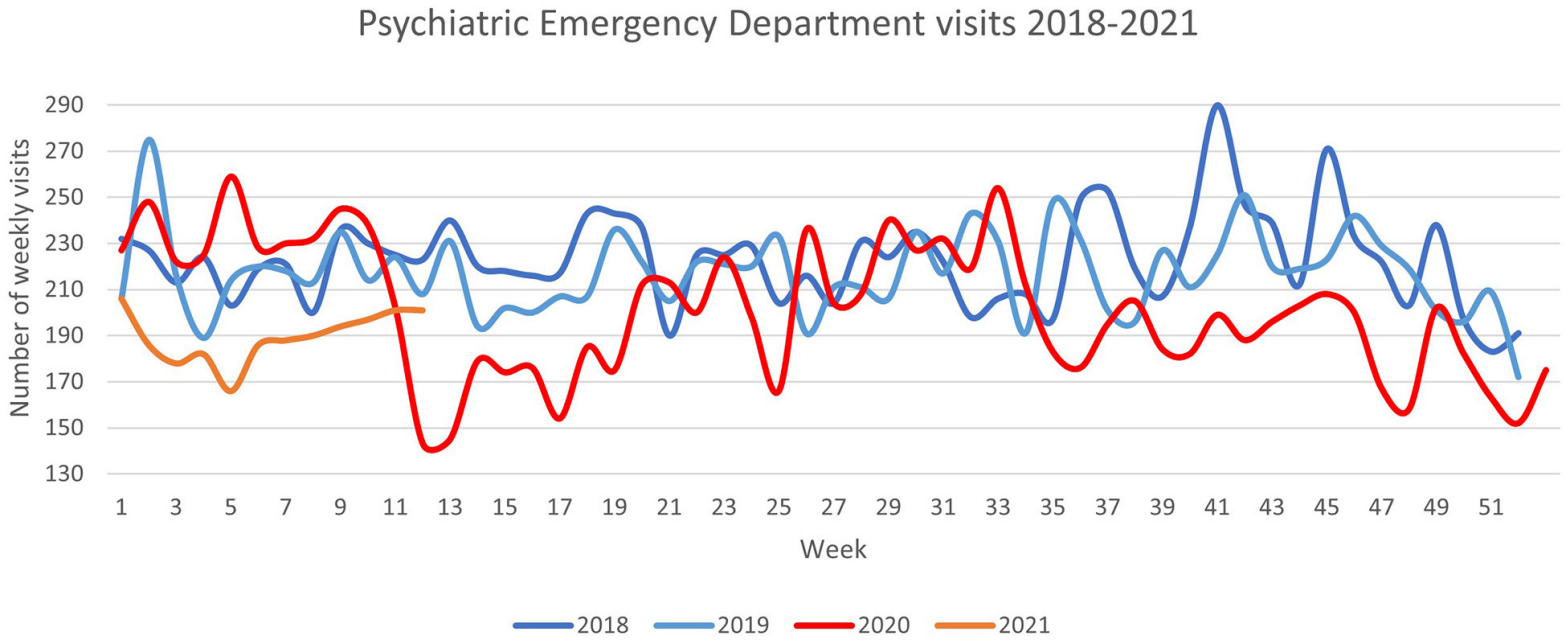
Number of visits per week to the Sahlgrenska University Hospital Psychiatric Emergency Department from 2018 to 2021.

The weekly number of visits to the Psychiatric Emergency Department during the study periods is illustrated in Figure 1. Table 1 shows the mean weekly number of PEVs during the COVID-19 periods and the control period overall and among patient subgroups according to demographic characteristics and psychiatric diagnoses. The mean weekly number of PEVs was significantly reduced by 16 and 15%, respectively, during the first and second waves of the COVID-19 pandemic compared to the pre-COVID-19 period. During the first COVID-19 wave, the largest reductions in mean weekly numbers of PEVs were seen in patients with unipolar and related mood disorders (38%) and substance abuse disorders (31%) as well as those not requiring ongoing outpatient care (22%). Statistically significant reductions in PEVs were also seen during the first COVID-19 wave in both sexes, in both older (≥65 years) and younger (18–64 years) individuals, among those with a diagnosis of substance abuse disorders, unipolar and related mood disorders, and neurotic, stress-related, and somatoform disorders, and those not requiring ongoing outpatient psychiatric care. However, there was a statistically significant increase of patients without a psychiatric diagnosis. There were no significant reductions in PEVs for patients with a diagnosis of schizophrenia and other related psychotic disorders, bipolar and related mood disorders, disorders of personality, and behavior in adults as well as those receiving ongoing care through one of the Sahlgrenska University Hospital's outpatient clinics. There was a similar pattern of significant PEV reductions (and non-significant results) among the different patient characteristic and diagnostic subgroups during the second COVID-19 wave as during the first wave, except for patients with bipolar and related mood disorders and patients with no diagnosis who did not show a significant reduction in PEVs during the second wave.

**Table 1:** Change in number of weekly patient visits to the Sahlgrenska University Hospital Psychiatric Emergency Department during the first and second COVID-19 waves compared to pre-COVID 19 control period: overall, by patient demographic characteristics and psychiatric diagnosis, and ongoing outpatient care.

**Variable**	**Control period**	**First COVID-19 wave**			**Second COVID-19 wave**		
	**Mean no. of**	**Mean no. of**	**Change vs**.	***p*-value for**	**Mean no. of**	**Change vs**.	***p*-value for**
	**patients/week**	**patients/week**	**control**	**change vs**.	**patients/week**	**control period**	**change vs**.
	**(95% CI)**	**(95% CI)**	**period**	**control period**	**(95% CI)**	**period**	**control period**
Total	218.1 (214.3–222.0)	184.3 (172.3–196.2)	−16%	<0.001	185.7 (175.4–196.0)	−15%	<0.001
Sex							
Male	112.4 (109.9–114.5)	90.9 (83.4–98.5)	−19%	<0.001	94.5 (84.7–100.7)	−16%	<0.001
Female	106.0 (103.5–108.4)	93.4 (85.4–101.3)	−12%	0.002	91.2 (84.7–97.7)	−14%	<0.001
Age							
18–64 years	194.7 (191.0–198.3)	164.9 (153.1–176.8)	−15%	<0.001	166.5 (156.9–176.2)	−14%	<0.001
≥65 years	22.3 (21.4–23.2)	18.7 (15.8–21.6)	−16%	0.02	18.4 (16.0–20.8)	−17%	0.002
Psychiatric diagnosis							
Substance use disorders	42.3 (40.9–43.6)	29.3 (25.0–33.6)	−31%	<0.001	30.9 (27.4–34.4)	−27%	<0.001
Schizophrenia and other related psychotic disorders	17.2 (16.5–17.9)	15.0 (12.7–17.3)	−13%	0.058	15.4 (13.5–17.2	−11%	0.053
Bipolar and related mood disorders	8.3 (7.8–8.8)	7.1 (5.4–8.8)	−14%	0.18	6.8 (5.4–8.2)	−19%	0.03
Unipolar and related mood disorders	20.7 (19.8–21.7)	12.8 (9.7–15.9)	−38%	<0.001	14.8 (12.3–17.3)	−28%	<0.001
Neurotic, stress-related, and somatoform disorders	53.2 (51.5–54.9)	40.9 (35.3–46.4)	−23%	<0.001	36.5 (31.9–41.0)	−31%	<0.001
Disorders of personality and behavior in adult persons	12.6 (12.0–13.1)	12.1 (10.3–14.0)	−3%	0.67	11.6 (10.1–13.2)	−7%	0.24
None	3.7 (2.8–4.5)	9.2 (6.5–12.0)	149%	<0.001	5.7 (3.5–8.0)	55%	0.08
Ongoing outpatient care							
Yes	76.2 (74.5–77.8)	72.4 (67.0–77.7)	−5%	0.16	74.1 (69.7–78.5)	−3%	0.35
No	116.0 (113.5–118.5)	90.5 (82.5–98.5)	−22%	<0.001	89.7 (83.1–96.3)	−23%	<0.001

*CI, confidence interval*.

### Admission Rates to Psychiatric Inpatient Care

In 2020, 31% of the PEVs resulted in hospital admission compared to 33% in 2019 and 38% in 2018. Table 2 presents the comparison of the hospital admission rates overall and according to patient demographic characteristics and psychiatric diagnoses during the two COVID-19 waves compared to the control period. Overall, there were no significant reductions in hospital admission rates during both COVID-19 waves compared to the control period. There were no significant reductions in hospital admission rates in the different subgroups according to patient demographic characteristics and psychiatric diagnoses during the first COVID-19 wave. During the second COVID-19 wave, there were significant reductions in admission in patients aged ≥65 years and in subgroups with diagnoses of substance abuse disorders. Furthermore, there was a statistically significant increase of patients with disorders of personality and behavior in adult persons.

**Table 2:** Change in weekly number of patients admitted to inpatient care to the Sahlgrenska University Hospital Psychiatric Emergency Department during the first and second COVID-19 waves compared to pre-COVID 19 control period: overall and by patient demographic characteristics and psychiatric diagnosis.

**Variable**	**Control period**	**First COVID-19 wave**			**Second COVID-19 wave**		
	**Mean no. of**	**Mean no. of**	**Change vs**.	***p*-value for**	**Mean no. of**	**Change vs**.	***p*-value for**
	**patients/week**	**patients/week**	**control**	**change vs**.	**patients/week**	**control period**	**change vs**.
	**(95% CI)**	**(95% CI)**	**period**	**control period**	**(95% CI)**	**period**	**control period**
Total	111.9 (109.3–114.6)	106.3 (97.6–115.0)	−5%	0.20	106.8 (99.7–113.9)	−5%	0.16
Sex							
Male	57.1 (55.5–58.7)	54.4 (49.3–59.5)	−5%	0.31	53.5 (49.3–57.7)	−6%	0.09
Female	54.8 (53.2–56.5)	51.9 (46.4–57.3)	−5%	0.28	53.3 (48.9–57.8)	−3%	0.50
Age							
18–64 years	91.8 (89.4–94.1)	87.8 (80.2–95.4)	−4%	0.30	89.9 (83.7–96.1)	−2%	0.55
≥65 years	18.9 (18.1–19.7)	18.1 (15.5–20.8)	−4%	0.59	16.3 (14.1–18.4)	−14%	0.02
Psychiatric diagnosis							
Substance use disorders	42.2 (40.8–43.6)	39.1 (34.6–43.7)	−7%	0.19	37.8 (34.1–41.5)	−10%	0.02
Schizophrenia and other related psychotic disorders	18.7 (17.8–19.6)	18.9 (15.9–21.9)	1%	0.87	19.1 (16.6–21.6)	2%	0.74
Bipolar and related mood disorders	8.7 (8.2–9.3)	8.6 (6.7–10.4)	−2%	0.86	8.1 (6.6–9.6)	−7%	0.40
Unipolar and related mood disorders	17.1 (16.3–17.9)	15.3 (12.7–17.9)	−10%	0.19	15.6 (13.4–17.7)	−9%	0.17
Neurotic, stress-related, and somatoform disorders	25.3 (24.3–26.3)	25.4 (22.1–28.7)	0%	0.96	24.4 (21.7–27.1)	−4%	0.51
Disorders of personality and behavior in adult persons	11.9 (11.3–12.6)	13.7 (11.7–15.7)	15%	0.08	14.6 (13.0–16.3)	23%	<0.001

*CI, confidence interval*.

### Survey

Descriptive statistics are presented in Table 3 for each statement among the 67 respondents who completed parts or the whole study survey. Nearly all the respondents (95.5%) were <70 years of age, 49.3% were women, and a majority (58.2%) had ongoing contact with outpatient psychiatric care. In statements 1–5, well over half (67.9–87.0%) of respondents disagreed that COVID-19 had a negative impact on their mental health or that it affected their visit to the Psychiatric Emergency Department in any way. Notably, in statements 6 and 7, about a quarter of respondents agreed that either a doctor's assessment in primary care or a video call with a doctor in psychiatry could have replaced their visit. Statements 8–14 concerned perceived patient safety and treatment during the pandemic: a majority of the respondents did not feel that patient safety or treatment was jeopardized. Furthermore, statements 15–17 showed that most of the respondents did not experience any discrimination in treatment even if they had symptoms compatible with COVID-19.

**Table 3:** Survey results among patients seeking care at Sahlgrenska University Hospital Psychiatric Emergency Department from November to December 2020 (*N* = 69).

**Statement**	**No. of patients**	**Response^a^**			**Mean response^a^ score (95% CI)**
		**Disagree**	**Neutral**	**Agree**	
		**(score 1–2)**	**Neutral (score 3)**	**(score 4–5)**	
COVID-19 has had a negative effect on my mental health	57	70.2	10.5	19.3	1.5 (1.3–1.7)
COVID-19 contributed to my visit to the emergency department	54	83.3	5.6	11.1	1.3 (1.1–1.5)
COVID-19 delayed my visit to the emergency department	56	82.1	3.6	14.3	1.3 (1.1–1.5)
I delayed my visit in fear of being infected	54	87.0	3.7	9.3	1.2 (1.1–1.4)
I delayed my visit to not burden the health care system	53	67.9	15.1	17.0	1.5 (1.3–1.7)
I wouldn't have come here if I were offered a time quickly at reception or in primary care	52	61.5	13.5	25.0	1.6 (1.4–1.9)
A video call from a doctor in psychiatry could have replaced my visit	50	52.0	24.0	24.0	1.7 (1.5–2.0)
I was worried about getting infected during my visit at the emergency department	60	81.7	3.3	15.0	1.3 (1.1–1.5)
I felt safe from getting infected at the emergency department	57	28.1	12.3	59.6	2.3 (2.1–2.6)
My needs were de-prioritized due to the COVID-19 safety arrangements	55	81.8	7.3	10.9	1.3 (1.1–1.5)
I got the health care I needed despite COVID-19	51	17.6	15.7	66.7	2.5 (2.3–2.7)
The protective equipment used at the emergency department felt safe regarding risk of infection	55	12.7	10.9	76.4	2.6 (2.4–2.8)
To assess patients in containers outside the emergency department feels safe	53	17.0	20.8	62.2	2.5 (2.2–2.7)
I got help quickly at the emergency department	53	39.6	13.2	47.2	2.1 (1.8–2.3)
The staff's treatment was affected negatively because I had symptoms compatible with COVID-19	33^a^	97.0	0.0	3.0	2.3 (1.9–2.7)
The staff treated me well when I had symptoms compatible with COVID-19	15^b^	33.3	6.7	60.0	3.5 (3.3–3.8)
The staff was alert to recognize COVID-19 symptoms	44	9.1	4.5	86.4	2.8 (2.6–3.0)

a *Responses to the statements were scored on 5-point Likert scales (1 = strongly disagree to 5 = strongly agree), which were thereafter categorized into disagree (score 1–2), neutral (score 3), and agree (score 4–5)*.

b *Twenty-three patients not included as they did not have COVID-19 symptoms*.

*^c^Fourty two patients not included as they did not have COVID-19 symptoms*.

*CI, confidence interval*.

## Discussion

A significant reduction in overall PEVs was found during the first and second COVID-19 waves, but there was no significant reduction in numbers of hospital admissions. The survey data supported the finding that COVID-19 did not have a negative impact on the respondents' mental health, how they perceived patient safety, and how they were treated by health care workers at the Psychiatric Emergency Department.

The overall PEV reduction of 16% during the first COVID-19 wave and 15% during the second wave was substantial. This reduction, however, was not as extensive as in many other studies (8, 13, 14). For example, a 27% overall reduction in PEVs was observed at an urban hospital in Ontario (12) and a 56% reduction within 2 months during full lockdown in Italy (8).

When analyzing patient demographic and psychiatric diagnosis subgroups for the reduction in PEVs, the only factors which did not show a significant reduction during both COVID-19 waves were patients with a diagnosis of schizophrenia and other related psychotic disorders, bipolar and related mood disorders or those with disorders of personality and behavior in adult persons as well as individuals with ongoing follow-up through outpatient psychiatric care. Both sexes showed a significant reduction in PEVs, although there was a higher reduction in absolute numbers in men compared to women. Men had higher mean weekly PEVs than women during the pre-COVID-19 control period. PEVs among the younger and elderly age groups showed a significant reduction, with a reduction in absolute numbers similar to the absolute reduction in overall PEVs during the pandemic periods. The unaltered number of PEVs among patients with schizophrenia and other related psychotic disorders stands in contrast to a report by the National Board of Health and Welfare in Sweden where other Swedish regions showed an increased rate of visits among patients with this psychiatric diagnosis (12). On the other hand, there are studies that show similar results to ours with either no significant reduction in this patient group (15, 24) or a smaller absolute reduction compared to other diagnoses (11). The largest absolute reduction among the different psychiatric diagnoses was seen among patients with substance abuse disorders and those with neurotic, stress-related, and somatoform disorders. A recent study at another large Swedish hospital reported similar results in patients with unipolar and anxiety-related disorders but, contrary to our results, the number of contacts related to substance use disorders remained unaltered (15). The mean weekly PEVs for patients with no diagnosis were numerically small during all time periods. However, there was a pronounced, significant increase during both COVID-19 waves compared to control, which is most likely due to an increased triage to home referral by a doctor or specialized nurse.

The overall number of hospital admissions remained unaltered during each COVID-19 wave. However, when testing the patient demographic and psychiatric diagnosis subgroups, age ≥65 years as well as those with substance use disorders or disorders of personality and behavior in adult persons were associated with significant reductions during the second COVID-19 wave. A decrease in hospital admissions among patients with substance abuse disorders may have several explanations. Recent studies have shown similar results, with authors emphasizing, for example, increased drinking of alcohol at home and consequent decreased public intoxications as a contributing factor (25, 26).

When interpreting our study results, with the aim to investigate the changes in utilization of acute psychiatric services during the COVID-19 pandemic, with a focus on risk groups, there are two main perspectives we want to highlight. First, our results indicate that acute psychiatric care was accessible and did not decrease for the most severely ill patients during the pandemic periods. This is mainly based on unaltered PEV numbers among patients with schizophrenia and other related psychotic disorders, among those with a requirement for ongoing psychiatric care, and the unaltered frequency of hospital admissions. This is also substantiated by the study survey, which indicated that the majority of the respondents at the Psychiatric Emergency Department did not experience a negative impact on their mental health or receipt of health care due to the pandemic. It might be assumed that patients with ongoing outpatient care generally suffer from more severe psychiatric disorders, such as schizophrenia or other related psychotic disorders. Furthermore, suicide rates did not increase during 2020 in Sweden (27) and Pirkis and colleague (28) have reported similar results when analyzing suicide rates in 21 high- or middle-income countries during the first month of the COVID-19 pandemic. The second perspective is that our results might indicate that patients with mild or moderate psychiatric disorders who did not seek acute psychiatric care to the same extent as before the pandemic received help and treatment through other units such as primary care. One can only speculate whether this patient group, despite emergency situations such as a pandemic, can be handled in primary care or in a different way such as through online means.

The COVID-19 pandemic has forced a rapid increase in the use of online psychiatry (29–31). In accordance with this, the Swedish National Board of Health and Welfare estimates that video consultations in psychiatric outpatient care during the pandemic have doubled compared to 2019 (32). Furthermore, in our study survey, about a quarter of the patients agreed that a video call from a doctor in psychiatry could have replaced their visit to the Psychiatric Emergency Department. A majority of the respondents were, however, <70 years of age and it is reasonable to believe that younger people might be more adept at seeking health care regarding their mental illness in alternative ways such as using online means. The elderly may not have adapted to these online alternatives to the same extent. This might result in a considerably deferred need for psychiatric care, especially among the elderly after the COVID-19 pandemic (33). In general, online psychiatry is often featured as cost saving, time saving, and a contribution to increased access of care (34). This can be extremely beneficial in times of emergency, such as during a pandemic, when in-person meetings may not be possible. An Israeli study has reported that the attitude toward the use of online means for health care in times of emergency is found to be positive (35). However, there are concerns regarding the use of online psychiatry in situations within acute psychiatric care, such as psychosis, acute crisis, or with risk of self-harm (36). Future research is needed to evaluate the actual clinical use and implementation of online psychiatry during the COVID-19 pandemic.

Among the potential limitations of our study, it was not possible to determine the causality of our results due to broad global differences in psychiatric health care structure but also in strategies to mitigate the spread of the COVID-19 pandemic. Furthermore, the study survey had few respondents, and the answers were collected during a limited time-period, which limits the possibility to draw any conclusions from the survey data. Another limitation is that the ICD-10 diagnosis codes defined at the Psychiatric Emergency Department are somewhat arbitrarily due to doctors with different competence and because the diagnoses are defined in a psychiatric emergency setting where the organization and time perspective must be considered, which may affect interpretation and, consequently, the frequencies of the psychiatric diagnoses in our study. Furthermore, patients who were sent home with advice from a triage nurse did not receive any psychiatric diagnosis in the reporting system. It is therefore impossible to confirm whether these patients would have affected the study outcome.

Among the strengths of our study is that the Sahlgrenska University Hospital Psychiatric Emergency Department is one of Scandinavia's largest with an average of 13,000 visits per year, which contributes to high internal validity. Furthermore, this study collected register data 2 years prior to the pandemic, which also contributes to high internal validity. Another strength is that, in contrast to many other similar studies, both psychiatric emergency department and hospital admission rates were examined to provide a more complete picture of the alterations in acute psychiatric service utilization. Moreover, in parallel to the utilization change analysis, a survey analysis was conducted examining the alteration of care-seeking behavior during the COVID-19 pandemic, contributing to an understanding of the reasoning when seeking care at a psychiatric emergency department. This might be argued to be one of this study's main additions to the literature since differing patterns in visits need to be investigated in tandem with views and attitudes of treatment seekers if the changes are to be fully understood.

In conclusion, the COVID-19 pandemic led to an overall reduction of PEVs but unaltered hospital admission rates at one of the largest psychiatric emergency departments in Northern Europe. The unaltered PEVs among patients with a diagnosis of schizophrenia and other psychotic disorders or those requiring ongoing outpatient psychiatric care as well as unaltered hospital admission indicates that severely ill patients still had access to specialized psychiatric care during the ongoing pandemic and restrictions. The long-term effects of these changes in utilization of psychiatric emergency services warrants further investigation and follow-up studies.

## Data Availability Statement

The data that supports the findings of this study are available from the corresponding author on request subject to data protection laws.

## Ethics Statement

The studies involving human participants were reviewed and approved by Swedish Ethical Review Authority (2020-04222). Written informed consent for participation was not required for this study in accordance with the national legislation and the institutional requirements.

## Author Contributions

MD, MI, and SS were involved in the conceptualization of the study. MH, TY, HC, and OF developed the methodology. SS, MH, and HC curated the study data. HC conducted the formal analysis. MH and TY undertook administration of the project and wrote the first draft of the article. All authors were involved in the conduct of the study, review and editing of the article, and approved the submitted version.

## Funding

SS's research time was financed by grants from the Swedish state under the agreement between the Swedish government and the county councils, the ALF agreement (ALFGBG-786541). The funder had no influence over the conduct or reporting of the study.

## Conflict of Interest

The authors declare that the research was conducted in the absence of any commercial or financial relationships that could be construed as a potential conflict of interest.

## Publisher's Note

All claims expressed in this article are solely those of the authors and do not necessarily represent those of their affiliated organizations, or those of the publisher, the editors and the reviewers. Any product that may be evaluated in this article, or claim that may be made by its manufacturer, is not guaranteed or endorsed by the publisher.

## References

[1] World Health Organization. Rolling Updates on Coronavirus Disease (COVID-19). (2020). Available online at: https://www.who.int/emergencies/diseases/novel-coronavirus-2019/events-as-they-happen (accessed November 14, 2021).

[2] Galea S Merchant RM Lurie N. The mental health consequences of COVID-19 and physical distancing: the need for prevention and early intervention. JAMA Intern Med. (2020)180:817–8. 10.1001/jamainternmed.2020.1562 32275292

[3] Qiu J Shen B Zhao M Wang Z Xie B Xu Y. A nationwide survey of psychological distress among Chinese people in the COVID-19 epidemic: implications and policy recommendations. Gen Psychiatr. (2020) 33:e100213. 10.1136/gpsych-2020-100213 32215365 PMC7061893

[4] Mazza C Ricci E Biondi S Colasanti M Ferracuti S Napoli C. A nationwide survey of psychological distress among Italian people during the COVID-19 pandemic: immediate psychological responses and associated factors. Int J Environ Res Public Health. (2020) 17:3165. 10.3390/ijerph17093165 32370116 PMC7246819

[5] Iasevoli F Fornaro M D'Urso G Galletta D Casella C Paternoster M. Psychological distress in patients with serious mental illness during the COVID-19 outbreak and one-month mass quarantine in Italy. Psychol Med. (2021) 51:1054–6. 10.1017/S0033291720001841 32423496 PMC7261960

[6] Alexiou E Steingrimsson S Akerstrom M Jonsdottir I Ahlstrom L Finizia C. A survey of psychiatric healthcare workers' perception of working environment and possibility to recover before and after the first wave of COVID-19 in Sweden. Front Psychiatry. (2021) 12:770955. 10.3389/fpsyt.2021.770955 34912253 PMC8666504

[7] Kavaliunas A Ocaya P Mumper J Lindfeldt I Kyhlstedt M. Swedish policy analysis for Covid-19. Health Policy Technol. (2020) 9:598–612. 10.1016/j.hlpt.2020.08.009 32904437 PMC7455549

[8] Montalbani B Bargagna P Mastrangelo M Sarubbi S Imbastaro B De Luca GP. The COVID-19 outbreak and subjects with mental disorders who presented to an Italian psychiatric emergency department. J Nerv Ment Dis. (2021) 209:246–50. 10.1097/NMD.0000000000001289 33214387

[9] Ambrosetti J Macheret L Folliet A Wullschleger A Amerio A Aguglia A. Impact of the COVID-19 pandemic on psychiatric admissions to a large Swiss emergency department: an observational study. Int J Environ Res Public Health. (2021) 18:1147. 10.3390/ijerph18031174 33525740 PMC7908206

[10] Hoyer C Ebert A Szabo K Platten M Meyer-Lindenberg A Kranaster L. Decreased utilization of mental health emergency service during the COVID-19 pandemic. Eur Arch Psychiatry Clin Neurosci. (2021) 271:377–9. 10.1007/s00406-020-01151-w 32519205 PMC7282463

[11] Gonçalves-Pinho M Mota P Ribeiro J Macedo S Freitas A. The impact of COVID-19 pandemic on psychiatric emergency department visits – a descriptive study. Psychiatr Q. (2021) 92:621–31. 10.1007/s11126-020-09837-z 32839923 PMC7445073

[12] Socialstyrelsen. Psykiatriska akutmottagningar har fortfarande färre besök men samtidigt fler inläggningar under coronapandemin. Available online at: https://www.socialstyrelsen.se/globalassets/sharepoint-dokument/artikelkatalog/ovrigt/2020-12-7076.pdf (accessed November 14, 2021).

[13] Kim HK Carvalho AF Gratzer D Wong AHC Gutzin S Husain MI. The impact of COVID-19 on psychiatric emergency and inpatient services in the first month of the pandemic in a large urban mental health hospital in Ontario, Canada. Front Psychiatry. (2021) 12:563906. 10.3389/fpsyt.2021.563906 33967842 PMC8102788

[14] Gómez-Ramiro M Fico G Anmella G Vázquez M Sagué-Vilavella M Hidalgo-Mazzei D. Changing trends in psychiatric emergency service admissions during the COVID-19 outbreak: report from a worldwide epicentre. J Affect Disord. (2021) 282:26–32. 10.1016/j.jad.2020.12.057 33387743 PMC7765763

[15] Håkansson A Grudet C. Decreasing psychiatric emergency visits, but stable addiction emergency visits, during COVID-19 – a time series analysis 10 months into the pandemic. Front Psychiatry. (2021) 12:664204. 10.3389/fpsyt.2021.664204 34326783 PMC8313759

[16] Clerici M Durbano F Spinogatti F Vita A de Girolamo G Micciolo R. Psychiatric hospitalization rates in Italy before and during COVID-19: did they change? An analysis of register data. Ir J Psychol Med. (2020) 37:283–90. 10.1017/ipm.2020.29 32368994 PMC7264453

[17] Boldrini T Girardi P Clerici M Conca A Creati C Di Cicilia G. Consequences of the COVID-19 pandemic on admissions to general hospital psychiatric wards in Italy: reduced psychiatric hospitalizations and increased suicidality. Prog Neuropsychopharmacol Biol Psychiatry. (2021) 110:110304. 10.1016/j.pnpbp.2021.110304 33737215 PMC8569419

[18] Ferrando SJ Klepacz L Lynch S Shahar S Dornbush R Smiley A. Psychiatric emergencies during the height of the COVID-19 pandemic in the suburban New York City area. J Psychiatr Res. (2021) 136:552–9. 10.1016/j.jpsychires.2020.10.029 33158555 PMC7992036

[19] Maripuu M Bendix M Öhlund L Widerström M Werneke U. Death associated with coronavirus (COVID-19) infection in individuals with severe mental disorders in Sweden during the early months of the outbreak – an exploratory cross-sectional analysis of a population-based register study. Front Psychiatry. (2021) 11:609579. 10.3389/fpsyt.2020.609579 33488430 PMC7819873

[20] Carlsen HK Oudin A Steingrimsson S Oudin Åström D. Ambient temperature and associations with daily visits to a psychiatric emergency unit in Sweden. Int J Environ Res Public Health. (2019) 16:286. 10.3390/ijerph16020286 30669579 PMC6352115

[21] Widgren BR Jourak M. Medical Emergency Triage and Treatment System (METTS): a new protocol in primary triage and secondary priority decision in emergency medicine. J Emerg Med. (2011) 40:623–8. 10.1016/j.jemermed.2008.04.003 18930373

[22] Folkhalsomyndigheten. Covid-19. (2021). Available online at: https://www.folkhalsomyndigheten.se/smittskydd-beredskap/utbrott/aktuella-utbrott/covid-19/ (accessed November 14, 2021).

[23] R Core Team. The R Project for Statistical Computing. (2020). Available online at: https://www.r-project.org/ (accessed November 14, 2021).

[24] Jagadheesan K Danivas V Itrat Q Shekaran L Lakra V. A 6-month study on the pattern of emergency department presentations for schizophrenia and other psychotic disorders during COVID-19 lockdown. Psychiatry Res. (2021) 303:114081. 10.1016/j.psychres.2021.114081 34246006 PMC8520318

[25] McAndrew J O'Leary J Cotter D Cannon M MacHale S Murphy KC. Impact of initial COVID-19 restrictions on psychiatry presentations to the emergency department of a large academic teaching hospital. Ir J Psychol Med. (2020) 38:108–15. 10.1017/ipm.2020.115 32996441 PMC7711497

[26] Herring AA Kalmin M Speener M Goodman-Meza D Snyder H Campbell A. Sharp decline in hospital and emergency department initiated buprenorphine for opioid use disorder during COVID-19 state of emergency in California. J Subst Abuse Treat. (2021) 123:108260. 10.1016/j.jsat.2020.108260 33612194 PMC7832157

[27] Socialstyrelsen. Statistik om dödsorsaker år 2020. (2021). p. 5.

[28] Pirkis J John A Shin S DelPozo-Banos M Arya V Analuisa-Aguilar P. Suicide trends in the early months of the COVID-19 pandemic: an interrupted time-series analysis of preliminary data from 21 countries. Lancet Phychiatry. (2021) 8:579–88. 10.1016/S2215-0366(21)00091-2 33862016 PMC9188435

[29] Corruble E. A viewpoint from Paris on the COVID-19 pandemic: a necessary turn to telepsychiatry. J Clin Psychiatry. (2020) 81:20com13361. 10.4088/JCP.20com13361 32237302

[30] Kannarkat JT Smith NN McLeod-Bryant SA. Mobilization of telepsychiatry in response to COVID-19 – moving toward 21^st^ century access to care. Adm Policy Ment Health. (2020) 47:489–91. 10.1007/s10488-020-01044-z 32333227 PMC7180652

[31] Liu S Yang L Zhang C Xiang YT Liu Z Hu S. Online mental health services in China during the COVID-19 outbreak. Lancet Psychiatry. (2020) 7:e17–8. 10.1016/S2215-0366(20)30077-8 32085841 PMC7129099

[32] Socialstyrelsen. Psykiatriska tillstånd och psykofarmaka under coronapandemin. Stockholm: Socialstyrelsen (2020).

[33] Vård för psykisk ohälsa under covid-19-pandemin – Stockholm, mars till september 2020. Centrum för epidemiologi och samhällsmedicin. Rapport (2021). p. 3. Available online at: https://www.folkhalsoguiden.se/globalassets/verksamheter/forskning-och-utveckling/centrum-for-epidemiologi-och-samhallsmedicin/folkhalsoguiden/rapporter-och-faktablad/rapport-2021.3-vard-for-psykisk-ohalsa-under-covid-19-pandemin.pdf (accessed November 14, 2021).

[34] Shore JH. Telepsychiatry: videoconferencing in the delivery of psychiatric care. Am J Psychiatry. (2013) 170:256–62. 10.1176/appi.ajp.2012.12081064 23450286

[35] Hamlin M Steingrimsson S Cohen I Bero V Bar-Tl A Adini B. Attitudes of the public to receiving medical care during emergencies through remote physician-patient communications. Int J Environ Res Public Health. (2020) 17:5236. 10.3390/ijerph17145236 32698481 PMC7400122

[36] Cowan KE McKean AJ Gentry MT Hilty DM. Barriers to use of telepsychiatry: clinicians as gatekeepers. Mayo Clin Proc. (2019) 94:2510–23. 10.1016/j.mayocp.2019.04.018 31806104

